# Non-coding RNA dysregulation in the amygdala region of schizophrenia patients contributes to the pathogenesis of the disease

**DOI:** 10.1038/s41398-017-0030-5

**Published:** 2018-02-02

**Authors:** Yichuan Liu, Xiao Chang, Chang-Gyu Hahn, Raquel E. Gur, Patrick A. M. Sleiman, Hakon Hakonarson

**Affiliations:** 1 Center for Applied Genomics, The Children’s Hospital of Philadelphia, Philadelphia, PA USA; 2 Neuropsychiatric Signaling Program, Department of Psychiatry, Perelman School of Medicine, University of Pennsylvania, Philadelphia, PA USA; 3 Neuropsychiatry Section, Department of Psychiatry, Perelman School of Medicine, University of Pennsylvania, Philadelphia, PA USA; 4 Division of Human Genetics, Department of Pediatrics, The Perelman School of Medicine, University of Pennsylvania, Philadelphia, PA USA

## Abstract

Schizophrenia (SCZ) is a neuropsychiatric disorder with a complex genetic etiology. The redundancy of the gene networks underlying SCZ indicates that many gene combinations have the potential to cause a system dysfunction that can manifest as SCZ or a related neurodevelopmental disorder. Recent studies show that small non-coding microRNA (miRNA) and long non-coding RNA (lncRNA) are important factors in shaping these networks and are dynamically regulated by neuronal activation. We investigated the genome-wide transcription profiles of 46 human amygdala samples obtained from 22 SCZ patients and 24 healthy controls. Using RNA sequencing (RNA-seq), we determined lncRNA expression levels in all samples and generated miRNA profiles for 27 individuals (13 cases and 14 controls). Previous studies have identified differentially expressed miRNAs in SCZ, including miR-132, miR-212, and miR-34a/miR-34c. Here we report differential expression of a novel miRNA, miR1307, in SCZ. Notably, miR1307 maps to a locus previously associated with SCZ through GWAS. Additionally, one lncRNA that was overexpressed in SCZ, AC005009.2, also maps to a region previously associated with SCZ based on GWAS and overlapped SCZ-related genes. The results were replicated in a large independent data set of 254 dorsolateral prefrontal cortex samples from the CommonMind consortium. Taken together, these results suggest that miRNA and lncRNAs are important contributors to the pathogenesis of SCZ.

## Introduction

Schizophrenia (SCZ) is a chronic, severe disabling neurodevelopmental disorder that affects people worldwide. While the exact cause of the disorder remains unknown, it is believed to result from an interplay between genetic and environmental factors^1, 2^. Recent genetic studies have implicated multiple genomic variants in functional gene networks that individually confer modest risk to the disease rather than high-impact mutations in single genes with large effect sizes^3–6^. Maintaining optimal network homeostasis requires coordination by an elaborate intracellular network of molecular signal transduction systems, including microRNAs (miRNAs), which are involved in the regulation and expression of genetic networks. Therefore, in addition to gene-specific variants that may affect an individual gene or pathway manifesting as SCZ or a related neurodevelopmental disorder^7, 8^, miRNAs such as non-coding RNAs that have the potential to dysregulate larger networks of genes have been postulated to be causal factors in the pathogenesis of SCZ^9^. Two groups of miRNAs, including typical non-coding RNAs and long non-coding RNAs (lncRNAs), have been reported to be associated with SCZ as well as other psychiatric diseases^7,10, 11^.

The amygdala dysfunction has been implicated in SCZ^12–14^; anatomically it consists of two almond-shaped groups of nuclei located deep and medially within the temporal lobes of the human brain. Functionally, the amygdala is thought to play a primary role in the processing of memory, decision-making, and emotional reactions^15^. Studies in mouse models involving the amygdala suggest that overexpression of miRNAs can protect the animals against anxiety-associated responses^16^ and controls their fear response^17^. Research in human subjects also implicates the amygdala region in emotion processing and recognition of facial expressions^18–20^. To our knowledge, a genome-wide transcription study of non-coding RNAs in the amygdala of SCZ patients has not been previously reported. In this study, we used next-generation sequencing technologies, including RNA sequencing (RNA-seq) and small RNA-seq (SRNA-seq), to assess the expression levels of miRNAs and lncRNAs in postmortem tissue from 46 individuals, including 22 SCZ patients and 24 healthy controls, obtained from the Lieber Institute. Our replication set consisted of 254 dorsolateral prefrontal cortex (DLPFC) tissues obtained from the DLPFC. Data that were generated as part of the CommonMind Consortium^21^ were of European ancestry.

## Materials and methods

### RNA extraction and quality assessment

Total RNA including the miRNA portion from postmortem amygdala tissue was extracted from 100 mg of pulverized tissue with all prep DNA/RNA/miRNA Universal Kit (Qiagen). The RNA portion was purified with RNeasy Mini Spin columns and on-column DNase digestion by RNase-Free DNase Set (Qiagen). Total RNA yield was determined by Qubit RNA BR Assay Kit and Qubit Fluorometer (ThermoFisher Scientific). RNA quality was assessed with high-resolution capillary electrophoresis on an Agilent Bioanalyzer 2100 (Agilent Technologies). Approximately 300 ng of total RNA was applied to an RNA 6000 Nano LabChip without prior heating. An RIN, obtained from entire Agilent electrophoretic trace with the RIN software algorithm, was used for the assessment of RNA quality (scale 1–10, with 1 being the lowest and 10 being the highest RNA quality).

### Small RNA library preparation and sequencing

The Illumina TruSeq® Small RNA kit was used to generate the small RNA-seq libraries according to the manufacturers’ instructions. Briefly, 3′ (RA3) and 5′ (RA5) adapters were sequentially ligated to Dicer-processed miRNAs. Adaptor-ligated small RNAs were reverse-transcribed and the resultant cDNAs PCR-amplified. The PCR products were size-separated on 2% agarose gels and the 22–30 nucleotide bands excised and purified. Purified products were quantified by qPCR before cluster generation on a cBot (Illumina). Twelve samples were multiplexed per lane and sequenced on an Illumina HiSeq2000at 1 × 36 or 1 × 50 bp.

### mRNA library preparation and sequencing

RNA-seq libraries were constructed using illumina TruSeq RNA sample Prep Kit (RS-122-2001) TruSeq RNA Sample Prep Kit V2, Illumina Inc., San Diego, CA, 92122) following the manufacturers’ instructions. The poly-A containing mRNA molecules were purified from 300 to 500 ng DNAse-treated total RNA using oligo (dT) beads. Following the purification, the mRNA was fragmented using divalent cations under elevated temperature (94 degree) for 2 min. Under these conditions, the resultant fragment lengths ranged from 130 to 290 bp with a median length of 185 bp. Reverse transcriptase and random primers were used to generate the first-strand cDNA from the RNA fragments. Second-strand DNA was synthesized using DNA polymerase I and RNaseH. The resulting cDNAs were end-repaired using T4 DNA polymerase, T4 PNK, and Klenow DNA polymerase and a single “A” base added using Klenowexo (3′–5′ exo minus). Illumine PE adapters were ligated using T4 DNA ligase including a barcoding index for multiplexing. These products were then purified and enriched by PCR to create the final cDNA library for high-throughput sequencing on a Highseq2000. The concentration of the RNA-seq libraries was determined by Qubit (Invitrogen, CA) and qPCR, and the quality of the libraries assayed on a Lab ChipGX (Caliper, MA) HT DNA 1 K/12 K/Hi-sensitivity LabChip. The libraries were multiplexed and loaded on a flow cell for cluster generation on a cBot (Illumina). Illumina Real Time Analysis was used for image analysis and base calling. FASTQ files were generated using the BCL Converter (CASAVA v1.8.2).

### RNA-seq/small RNA-seq alignment and differential expression test

Following sequencing we obtained an average read depth of 116 M reads per sample, ranging from 34 to 226 M reads across the 46 amygdala samples. Alignments were generated using the Genomic Short-read Nucleotide Alignment Program^22^ taking common SNPs (dbSNP137) into account during the alignments. The output SAM files were converted to BAM, sorted by index, and unpaired reads were filtered with SAMtools^23^. Following alignment and filtering an average of 103 M reads were obtained per sample with an average mapping/filtering rate of 89% (Supplementary Table 1). Differential expression tests were performed using the *cuffdiff* package in cufflink2.2.1^24^ and the GTF file from GENCODE version 19^25^. The DLPFC RNA-seq data were processed using the same bioinformatics pipeline as previously described for the amygdala data. To reduce algorithm-specific false-positives and to adjust for clinical covariates, we also analyzed the data set using DEseq2 including age, gender, postmortem index (PMI), and manner of death as covariates in the model. Differentially expressed non-coding RNAs were identified from the intersect of the Cuffdiff and DEseq2^26^ data sets.

The small RNA-seq data read depths ranged from 4.7 to 72 M with average 20 M reads per sample. Cutadapt1.8.1 was applied to trim the adaptors^27^ and the trimmed data were further aligned with Micro Read Fast Alignment Search Tool (MRFast)^28^. The average mapping rate after trimming was 75.1% with an average read depth of 16 M reads per sample.

### Clinical sample selection and sample data QC

A detailed description of the clinical sample is provided in Supplementary Table 6. RNA-seq and small RNA data were generated on all 49 individuals listed in Supplementary Table 6. Following primary analysis of the RNA-seq data, three individuals (10451, 10678, and 10495) were excluded from further analysis because of outlying expression values. The five most highly differentially expressed genes (*CCL3, CCL4, CCL8, CXCL10, and CXCL11*) in the analysis were driven by these three controls, with extreme fragments per kilobase million (FPKM) values (~×100 higher than the other samples). The outliers may be related to the cause of death of these individuals, two (10451, 10678) died from “complications of smoke inhalation”, and the third (10495) from an accident with multiple head injuries. Of the remaining 46 cases and controls, all were of Caucasian ancestry, 40 were males, and 6 females. Average age at death was 38.4 years. As confounders are an important consideration in postmortem studies, we carried out non-parametric analyses to test for association between age, sex, and PMI with SCZ and gene expression level (FPKM) in the remaining 46 samples. No significant associations were found between FPKM or SCZ and the covariates. Following quality control of the small RNA-seq data, 19 of the 46 individuals with RNA-seq data were excluded from the analysis.

In validation data set DLPFC, RNA was isolated from 50 mg homogenized tissue in Trizol using the RNeasy kit based on the instructional protocol. The mean total RNA yield was 15.3 µg (±5.7). The RNA integrity number (RIN) was determined by fractionating RNA samples on the 6000 Nano chip (Agilent Technologies) on the Agilent 2100 Bioanalyzer. The mean RIN was 7.7 (±0.9), and the mean ratio of 260/280 was 2.0 (±0.02). Processing order was re-randomized prior to ribosomal RNA (rRNA) depletion. Briefly, rRNA was depleted from about 1 µg of total RNA using Ribo-Zero Magnetic Gold kit (Illumina/Epicenter Cat # MRZG12324) to enrich for polyadenylated coding RNA and non-coding RNA. The sequencing library was prepared using the TruSeq RNA Sample Preparation Kit v2 (RS-122–2001-48 reactions) in batches of 24 samples. A pool of 10 barcoded libraries was layered on a random selection of two of the eight lanes of the Illumina flow cell bridge amplified to ~250 million raw clusters. One-hundred base pair paired end reads were obtained on a HiSeq 2500. The sequence data were processed for primary analysis to generate QC values. Samples with a minimum of 50 million mapped reads (~25 million paired end reads) and less than 5% rRNA-aligned reads were retained for downstream analysis^21^.

## Results

### Replication of known SCZ-related miRNA from the small RNA-seq data

The small RNA-seq data set included 27 individuals (13 cases and 14 controls); differential expression analysis resulted in the identification of 17 statistically significant differentially expressed miRNAs (Table 1). Ten of the seventeen are novel discoveries, the remaining seven have previously been implicated in SCZ and/or other psychiatric phenotypes, including two members of miRNA family 34, mir-34c, -34a (*p* value = 3e−4 and 0.02, respectively) as well as a number of others including mirs-124,-132, -212, -663, and -144.

**Table 1:** MicroRNA differentially expressed based on small RNA-seq of 27 individuals

MicroRNA_ID	Locus	FPKM_SCZ	FPKM_controls	*p* value	Previous SCZ miRNA	Functions
hsa-mir-196a-2	chr12:54385521–54385631	90.3847	0	5.00E − 05	N	
hsa-mir-1975	chr7:148638579–148638654	6.93E + 06	3.38E + 06	0.00015	N	
hsa-mir-663	chr20:26188821–26188914	1352.72	3820.51	0.00015	Y	Suppresses the expression of multiple genes implicated in neurogenesis
hsa-mir-34c	chr11:111384163–111384240	777 765	346 426	0.0003	Y	Acute restraint stress in rodents were observed in the central amygdala, increasing expression of hsa-mir-34c introduce to anxiety
hsa-mir-639	chr19:14640354–14640452	5.59116	87.504	0.0007	N	
hsa-mir-132	chr17:1953201–1953302	1.40E + 06	2.12E + 06	0.00235	Y	Bipolar in mouse; gene network in MDD patients; differentially expressed in human prefrontal cortex; potential biomarker for SCZ
hsa-mir-124-2	chr8:65291705–65291814	1046.85	2008.49	0.00655	Y	Link to anxiety through glucocorticoid signaling
hsa-mir-451	chr17:27188386–27188458	6.95E + 06	4.97E + 06	0.01085	N	
hsa-mir-212	chr17:1953564–1953674	58 273.3	91832	0.0125	Y	Differentially expressed in human prefrontal cortex; potential biomarker for SCZ
hsa-mir-483	chr11:2155363–2155439	2268.02	5351.61	0.01525	N	
hsa-mir-886	chr5:135416176–135416297	2773.91	5943.91	0.0226	N	
hsa-mir-34a	chr1:9211726–9211836	45 606.8	30 774.7	0.0234	Y	Control fear response in mice amygdala; differentially expressed in human prefrontal cortex. Potential biomarkers in blood for SCZ
hsa-mir-375	chr2:219866366–219866430	47 419.6	25 485.4	0.02615	N	
hsa-mir-585	chr5:168690604–168690698	3850.95	6422.48	0.03325	N	
hsa-mir-424	chrX:133680643–133680741	16 779	25 251.9	0.038	N	
hsa-mir-144	chr17:27188550–27188636	229 142	160 058	0.04165	Y	Dysregulated in hippocampus of rats by the mood stabilizers valproate and lithium
hsa-mir-520d	chr19:54223349–54223436	18.5836	89.2044	0.0489	N	

### miRNA based on RNA-seq

**Fig. 1:**
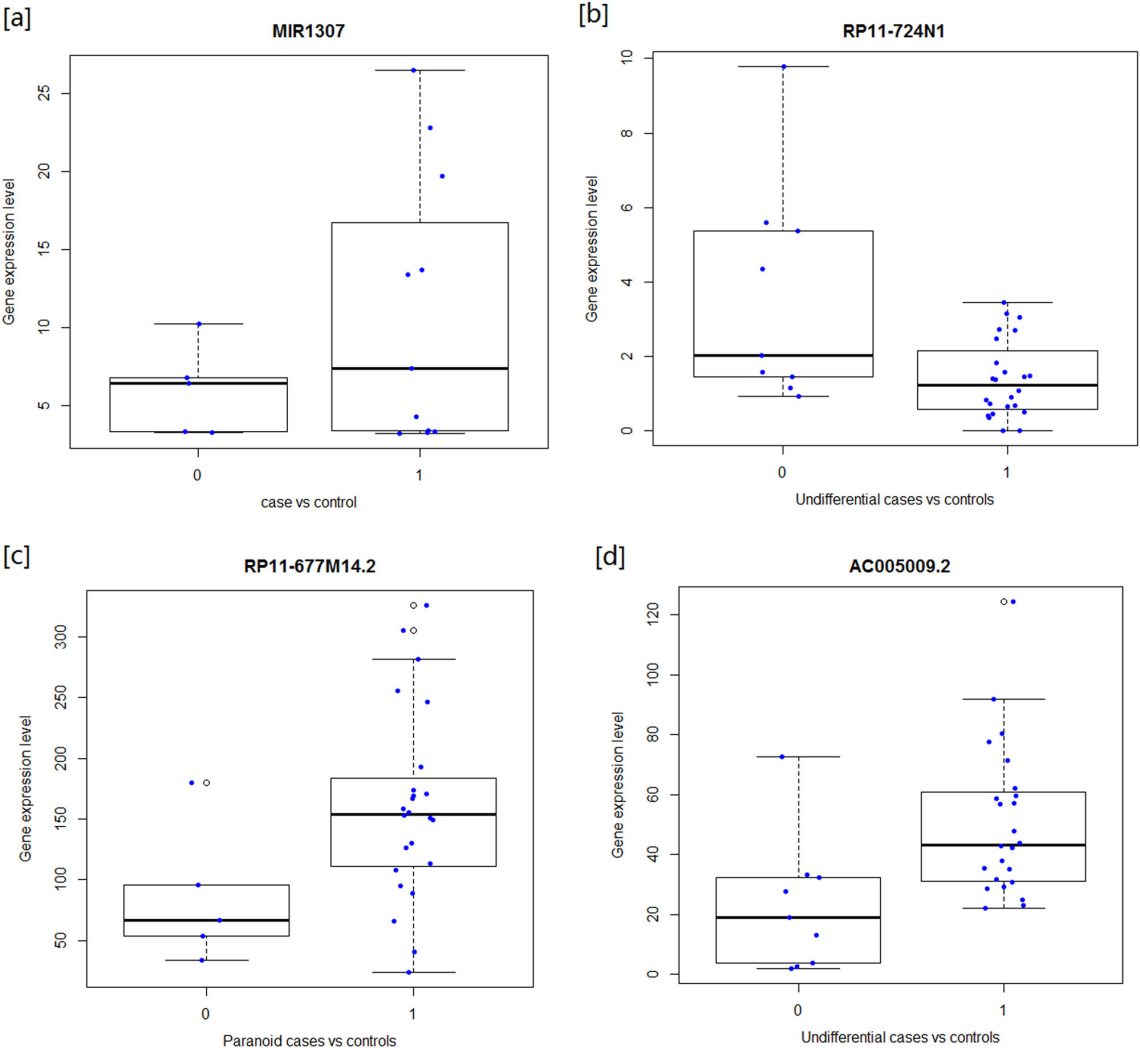
**Boxplot of target ncRNAs between SCZ/controls and SCZ subtypes** **a** There are 5 individuals in case has non-zero expression on MIR1307 and 11 individuals in controls. The mean of cases is 5.9 and 11 for controls. **b** For RP11-724N1.1, the mean gene expression in cases is 3.6 and in controls is 1.4; the overall expression is about three fold higher in SCZ cases. **c** The mean gene expression of RP11-677M14.2 is 86 in paranoid cases, which is highly driven by one outlier with expression 179; the mean gene expression in controls is 160. **d** The mean expression of AC005009.2 for undifferentiated cases is 23 and 51 in controls

While RNA-seq is not designed to systematically detect expression changes of miRNA, some miRNAs that are affiliated with the RNA class are detectable by RNA-seq. Analysis of the GTF file as described in the Materials and methods section resulted in the identification of differential expression of *MIR1307* between the SCZ patients and healthy controls. Expression of MIR1307 was approximately threefold higher in controls (*p* value = 0.0152). The expression difference and the direction of change were confirmed in the small RNA-seq data set, plotting the expression levels in cases and controls confirms that the association is not driven by outliers (Fig. 1a).

### LncRNAs based on RNA-seq

Differential analysis of the transcriptome data identified 250 lncRNAs from Gencode version 19 that showed significant expression differences between the amygdala regions of cases and controls (Supplementary Table 2). Among those, two lincRNAs* RP11-724N1.1* (*p* value = 0.014) and *RP11-677M14.2* (*p* value = 0.045) are of particular interest as they map to regions previously associated with SCZ based on genome-wide association study (GWAS) (Table 2). *RP11-724N1.1* resides within the GWAS locus on chr10, previously shown to be associated with SCZ in multiple studies^29, 30^. *RP11-677M14.2 is* located within the SCZ-associated gene, neurogranin (NRGN)^30^, the human homolog of the neuron-specific rat RC3/neurogranin gene, which is a direct target for thyroid hormone in the human brain, and controls its expression.

**Table 2:** Summary of lncRNAs associated with SCZ

LncRNA	RP11-724N1.1	RP11-677M14.2
Locus	chr10:104674341–104675161	chr11:124614529–124616233
FPKM_SCZ	2.6918	110.377
FPKM_control	1.41326	148.519
*p* value	0.014	0.04545
SCZ GWAS regions	chr10:104487871–105245420, chr10:104423800–105165583,	chr11:124610007–124620147,
SCZ SNPs	rs2297787 (*p* value = 1.53E−13; distance = 4976 bp), rs11191419 (*p* value = 9.24E−18; distance = 62k bp), rs55833108 (*p* value = 1.42E−8; distance = 66k bp)	rs55661361 (pV = 3.68E−12; distance = 578 bp)
Nearby genes	CNNM2	NRGN

### Non-coding RNAs for SCZ subtypes

We further categorized the SCZ patients based on their clinical subtypes, including disorganized (seven individuals), paranoid (five individuals), and undifferentiated (nine individuals; Supplementary Table 7). The analysis revealed subtype specificity in non-coding RNA expression levels. MIR1307 showed 10-fold higher expression in controls when compared with the undifferentiated subtype in contrast to a two-fold increase when compared with the paranoid subtype. MIR1282 showed significantly higher expression in the paranoid subtype. We also identified subtype specificity in the expression of miRNAs previously reported to be associated with SCZ or other psychiatric diseases. For instance, the miRNA family 34 were only significant in the undifferentiated subtype and mir-144 was only associated with the disorganized subtype (Supplementary Table 4). For lncRNAs, *RP11-677M14.2* was shown to be differentially expressed in the paranoid subtype (*p* value = 0.0064), whereas *RP11-724N1.1* was differentially expressed in undifferentiated SCZ (*p* value = 0.016; Supplementary Table 5). Finally, some associations were restricted to a particular subtype such as lncRNA *AC005009.2*, which was not differentially expressed in the combined analysis but is significantly associated with the undifferentiated subtype (*p* value 0.0016).

Plotting the expression value distributions of the three lncRNAs differentially expressed by subtype (Supplementary Figs. 2a–e) indicates that the results are not driven by outliers. To determine the effects of variables such as age, gender, and manner of death (Supplementary Fig. 3), we used expression values of target lncRNAs as dependent variable (y) and age, gender, and mode of death as independent variable (*x*). For RP11-724N1.1, the *p* value of linear regression is 0.312, 0.973, and 0.504 for age of death, gender, and manner of death. For RP11-677M14.2 the *p* values are 0.2735, 0.3565, and 0.0954, and for AC005009.2 the *p* values are 0.0429, 0.7158, and 0.7331, respectively. Therefore, the expression level differences of subtypes are not driven by the biological parameters at a significance level of *p* ≤ 0.01. The entire distribution difference *p* values of expressed lncRNAs based on Wilcoxon rank-sum between undifferentiated, paranoid, and disorganized are 0.056, 0.15, and 0.89. The statistic results indicate that the lncRNA distribution between undifferentiated and paranoid are close to significant difference, and there are certain level differences between paranoid and disorganized, but the differences between undifferentiated and disorganized are insignificant. As a result, it suggests that paranoid SCZ has unique lncRNA patterns compared to other subtypes.

The DESeq2 results of the three lncRNAs were highly concordant with the cuffdiff outputs, expression fold changes were consistent, and almost identical between two tools (Supplementary Table 8). The boxplot of those three lncRNAs showed that the expression differences are not driven by outliers (Figs. 1b–d).

### Replication/validation from independent data set

With limited availability of postmortem amygdala tissue from SCZ patients and no previously reported human amygdala RNA-seq studies available in the public domain, we attempted replication of our findings in DLPFC^21^, an independent data set from the CommonMind Consortium. RNA-seq was carried out in the DLPFC of totally 254 individuals (120 SCZ and 134 controls) of European ancestry. We applied the same bioinformatics analysis pipeline to the DLPFC data as previously described for the amygdala data to validate our lncRNA/miRNA findings. Adequate coverage was obtained for lncRNA *AC005009.2* in the replication set but not for the rest three non-coding RNAs. The read depth of DLPFC data set is about half that of the amygdala data at 60~70 M reads vs. 150 M reads. As shown in Table 3, *AC005009.2* replicated with a significant *p* value, and MIR1307 showed a clear trend toward association, with the same direction of effect, in the DLPFC data (Table 3).

**Table 3:** Replications of target lncRNAs/miRNA in dorsolateral prefrontal cortex tissues (DLPFC)

Tissue	LncRNA	Locus	Case	Control	pV
Amygdala	RP11-724N1.1	chr10:104674341–104675161	3.57426	1.37889	0.01605
DLPFC	RP11-724N1.1	chr10:104674341–104675161	1.81873	1.65285	NA (no enough reads)
Amygdala	RP11-677M14.2	chr11:124614529–124616233	85.7424	160.158	0.0064
DLPFC	RP11-677M14.2	chr11:124614529–124616233	4.01757	4.47271	0.27675
Amygdala	AC005009.2	chr7:86413541–86415986	22.9313	50.602	0.00165
DLPFC	AC005009.2	chr7:86413541–86415986	69.0017	92.058	0.0006
Amygdala	mir1307	chr10:105154009–105154158	1.36	4.08	0.0152
DLPFC	mir1307	chr10:105154009–105154158	5.16	8.18	NA (no enough reads)

## Discussion

SCZ is a complex polygenic disease. Multiple risk genes have already been identified through GWAS and sequencing studies^30, 31^. To date, no variants, common or rare, conferring large effect sizes have been identified in SCZ. Rather a picture is emerging from the genetic studies implicating the dysregulation of complex gene networks and regulatory mechanisms. Growing evidence indicates that distinct neuronal ncRNAs, particularly miRNAs and lncRNAs, are likely to influence the development of psychiatric diseases, including SCZ. For instance, the lncRNA, Gomafu, is acutely regulated in response to neuronal activation and is involved in SCZ-associated alternative splicing. In this study, we detected altered expression of miRNAs and lncRNAs, well defined in GENCODE annotation, by RNA-seq and SRNA-seq of amygdala tissue samples from 46 subjects including SCZ subjects and controls.

Amygdala dysfunction has been extensively reported in SCZ, yet to our knowledge, gene expression profiling has not been previously reported on human amygdala tissue. Previous studies in mice show that overexpression of miRNA 34 genes in amygdala is associated with anxiety and fear. Overexpression of mir-34c in the amygdala of mice has been shown to induce anxiolytic behavior after challenge^16^; in our data set, the expression of mir-34c was twofold higher in the SCZ group compared to the control. Mir-34a has been implicated in amygdala-dependent fear memory consolidation in mice through Notch signaling^17^ and in our data expression of mir-34a was significantly upregulated in cases. Mir-34a has been shown to be differentially expressed in other brain regions, including the prefrontal cortex^32^, leading to the suggestion that mir-34a levels in blood could serve as a biomarker for SCZ^33, 34^. In our study, we identify that the 34 family is only differentially expressed in the individuals with “undifferentiated” subtype of SCZ. Another example is mir-132, a well-known miRNA involved with circadian rhythm and previously associated with multiple psychiatric traits in mouse models^35^ and with network regulation in major depressive disorder in human prefrontal cortex tissue^36^. Mir-132 is also differentially expressed in human prefrontal cortex tissue in miRNA-profiling data^32^ and is a potential biomarker in human blood^33^. In our study, we show that mir-132 is differentially expressed across all of the clinical SCZ subtypes. Another miRNA of interest, mir-663, is a known regulator of neuronal differentiation^37^. Finally, mir-144 is expressed in the hippocampus of rats and is known to be influenced by both valproate and the mood stabilizer lithium^38^; mir-144 is found to be only differentially expressed in individuals categorized as “disorganized” SCZ.

In addition to the seven previously reported miRNAs, we further identified 10 miRNAs that will require validation in future human or mouse studies. We also identified over 200 differentially expressed lncRNAs in this study. In order to prioritize the lncRNAs, we focused on differentially expressed lncRNAs that mapped to previously reported GWAS loci. Two lncRNAs, *RP11-724N1.1* and *RP11-677M14.2*, mapped to GWAS loci at chr10q24.3 and chr11q24.2, respectively. *RP11-724N1.1* maps to the third most significantly associated region from the PGC2 SCZ meta-analysis (rs11191419, *p* value = 6.29 × 10^−19^)^39^. Similar to miRNAs, *RP11-724N1* is also clinical subtype-specific; it is only differentially expressed in undifferentiated SCZ subtype. *RP11-724N1.1* overlaps the *CNNM2* gene, as shown in Supplementary Fig. 1a.* CNNM2* encodes a brain-expressed transmembrane protein that is involved in magnesium transport^40^. Loss of function mutations in *CNNM2* has recently been reported to be causal of hypomagnesemia, seizures, and mental retardation^41^. *RP11-677M14.2* maps to the antisense strand overlapping the *NRGN* gene (Supplementary Fig. 1b). The *NRGN* locus has been shown to be associated with SCZ in the PGC2 meta-analysis (rs55661361, *p* value = 3.68E−12), as well as in multiple other studies that reported association of functional coding variants in *NRGN* with SCZ^39, 42–45^. Post-transcriptional regulation of *NRGN* through overlapping sense and antisense transcripts has previously been reported during cerebral corticogenesis and synapse function in mice^46^. We can confirm through our RNA-seq expression data (Supplementary Table 3) that the expression of *NRGN* is strikingly higher (FPKM = 1251) in the SCZ group compared to controls (FPKM = 0). Subtype analysis further indicated that *RP11-677M14.2* under expression was restricted to cases with a clinical diagnosis of paranoid SCZ.

A subtype-specific lncRNA, AC005009.2, has been identified for undifferentiated SCZ individuals; the lncRNA shows twofold higher expressions in control compared to SCZ patients who were diagnosed as the “undifferentiated” subtype. AC005009.2 is also close to GWAS region of PGC2 SCZ meta-analysis (rs12704290, *p* value = 1.04 × 10^−10^). Meanwhile, AC005009.2 overlaps the transcript of the metabotropic glutamate receptor 3 gene (GRM3; Supplementary Fig. 5c). *GRM3* has been shown to associate with SCZ risks in previous studies^47–49^, and AC005009.2 is only differentially expressed in undifferentiated SCZ subtype with *p* value = 0.0046 and 1.5-fold higher expression in controls.

*MIR1307* also maps to a highly significant SCZ GWAS locus (rs11191419, *p* value = 6.198e−19) in the PGC2 meta-analysis^30^. The associated locus contains multiple genes; however, we only detected differential expression in our RNA-seq data set from *MIR1307* (Table 4). The expression data raise the possibility that the GWAS association is being driven by *MIR1307*. Further work will be required to assess the association between the SCZ-associated GWAS variants and *MIR1307* expression levels in the brain.

**Table 4:** Gene expressions of SCZ GWAS region chr10:104487871–105245420

Categories	Gene ID	*p* value
Gene in relation to index SNP	C10orf32-AS3MT	na
	C10orf26	0.59655
	CALHM1	0.3712
	CALHM2	0.5112
	CALHM3	na
	CNNM2	0.7684
	CYP17A1	na
	INA	0.14265
Other genes in genomic region defined by LD	MIR1307	0.0152
	NT5C2	0.87125
	PCGF6	0.57665
	PDCD11	0.8658
	SFXN2	0.5662
	TAF5	0.9112
	USMG5	0.98425
	ACTR1A	0.96425
	ARL3	0.6029
	AS3MT	0.80155
eQTL	C10orf26	0.59655
	C10orf32	0.41385
	NT5C2	0.87125
	TMEM180	0.6154
	TRIM8	0.5002

“na” indicates there is not enough coverage for the gene

To validate and replicate our findings in the absence of an independent human amygdala RNA-seq data set, we turned to a much larger independent data set of 254 prefrontal cortex tissues. The involvement of the DLPFC in SCZ is well established with multiple reports of association of DLPFC dysfunction correlating with clinical phenotype^50, 51^. *AC005009.2* replicated with a significant *p* value and MIR1307 showed a clear trend toward association, with the same direction of effect, in the DLPFC data. The two other lncRNAs, *RP11-724N1.1 and RP11-677M14.2*, did not reach statistical significance in the DLFPC data, which may either be reflective of tissue specificity or simply due to the reduced coverage in the DLPFC data. The average read coverage in the DLPFC was 70 M reads compared with the 150 M reads from the amygdala data.

Finally, we note that several previously reported SCZ-associated miRNAs, including MIR137, 181b, 19, 219, and MIR9, did not replicate in this study. MIR137 and MIR181b were based on GWAS results rather than brain tissues, and MIR181b was reported in Chinese population rather than in Caucasian^52–54^. GWAS studies based on blood showed that MIR137 mediated dysregulation as a previously unknown etiologic mechanism in SCZ^52^. Our results showed that MIR137 expression is almost same in SCZ and controls, and MIR181b is 1.2-fold higher than SCZ. MIR19 was reported recently to have abnormal expression in neural progenitor cells from SCZ patient-derived induced pluripotent stem cells (iPSCs)^55^; in our data the expression of MIR19 was 1.2-fold higher in SCZ patients, but it did not reach significance (*p* value = 0.43). MIR219, a brain-specific miRNA, was reported in both normally developing and SCZ patient iPSC-derived neural stem cells^56^. In our amygdala data, the expression level of MIR219 was 1.4-fold higher in SCZ; however, it did not reach significance with a *p* value of 0.19. The association of those two miRNA was initially reported in SCZ NPCs but not in brain tissue, which may explain the lack of replication. MIR-9 was reported to be abundantly expressed in control neural progenitor cells (NPCs) and significantly downregulated in a subset of SCZ NPCs^57^. In Cuffdiff pipeline, the mir-9 was marked as region with too many fragments, so no *p* value was generated. In contrast, DESeq2 reports significant *q* value of mir-9 (0.02).

## Conclusion

Differential expression analysis of small RNAs in SCZ brain identified multiple genes that may be contributing to the pathogenesis of the disease. Intersecting the list of differentially expressed small and lncRNAs with loci identified in previously reported GWAS studies has highlighted two genes, miRNA (mir1307) and lncRNAs (AC005009.2), that are differentially expressed in SCZ and as such may underlie or contribute to the observed GWAS signals at these loci. This study demonstrates the utility of transcriptional profiling of relevant disease tissues in identifying genes underlying GWAS signals.

## Electronic supplementary material

Legends of supplementary materials

Alignment Statistic

Differentially expressed lncRNAs

Differentially expressed genes

miRNAs in SCZ subyptes

lncRNAs in SCZ subtypes

Phenotype of 49 individuals in the study

Patient ID for SCZ subtypes

Results comparisons between cuffdiff and DEseq2

Genome Browser Visualizations of lncRNAs and nearby genes

Boxplot of RP11-724N1.1 between undifferentiated and paranoid

Boxplot of RP11-677M14.2 between undifferentiated and paranoid

Boxplot of AC005009.2 between undifferentiated and paranoid

Boxplot of RP11-724N1.1 between undifferentiated and disorganized

Boxplot of AC005009.2 between disorganized and undifferentiated

Boxplot of RP11-677M14.2 between disorganized and paranoid

Age distribution of SCZ subtype patients

Death distribution of SCZ subtype patients
