# Supplementary material for: Non-coding RNA dysregulation in the amygdala region of schizophrenia patients contributes to the pathogenesis of the disease
Source: Transl Psychiatry. 2018 Feb 2;8:44. doi: 10.1038/s41398-017-0030-5 (PMC5804029; doi:10.1038/s41398-017-0030-5)
Supplement: Supplementary file 1 — Legends of supplementary materials [file 41398_2017_30_MOESM1_ESM.docx]

**Supplementary Figure 1.** Genome Browser Visualizations of lncRNAs and nearby

**Supplementary Figure 2.** Expression boxplots of RP11-724N1.1 , RP11-677M14.2 and AC005009.2 among three different SCZ subtypes (undifferentiated, paranoid and disorganized)

**Supplementary Figure 3.** Age and manner of death distribution of SCZ subtype patients. [a] The average ages at death for disorganized, paranoid and undifferentiated subtypes are 42.9, 39.2 and 46.1 respectively. [b] There are three mode of death in our data, including accident, suicide and natural. Every subtype contains all three modes of death.

**Supplementary Table 1.** RNA-seq/small RNA-seq alignment statistic.

The table contains the information of reads depth before/after alignments with SCZ/control annotation

**Supplementary Table 2.** Differentially expressed lncRNA list

**Supplementary Table 3.** Differentially expressed gene list

**Supplementary Table 4.** miRNAs expressions in SCZ subtypes

The table lists the differentially expressed miRNAs in SCZ subtypes, include disorganized, paranoid and undifferential

**Supplementary Table 5.** lncRNAs expressions in SCZ subtypes

The table lists the differentially expressed lncRNAs in SCZ subtypes, include disorganized, paranoid and undifferential

**Supplementary Table 6.** Clinical phenotype of 49 patients in the study.

Details of 49 individuals selected in this study, including age, sex, PMI, cause of death, clinical diagnosis and habit information

**Supplementary Table 7.** Patient ID in different SCZ subtype.

**Supplementary Table 8.** Differential expression tests for target non-coding RNAs between different computational tools.

Targets ncRNAs between Cuffdiff and DESeq2, the results are highly consistent to each other
