# Supplementary figures and images for: Non-coding RNA dysregulation in the amygdala region of schizophrenia patients contributes to the pathogenesis of the disease
Source: Transl Psychiatry. 2018 Feb 2;8:44. doi: 10.1038/s41398-017-0030-5 (PMC5804029; doi:10.1038/s41398-017-0030-5)

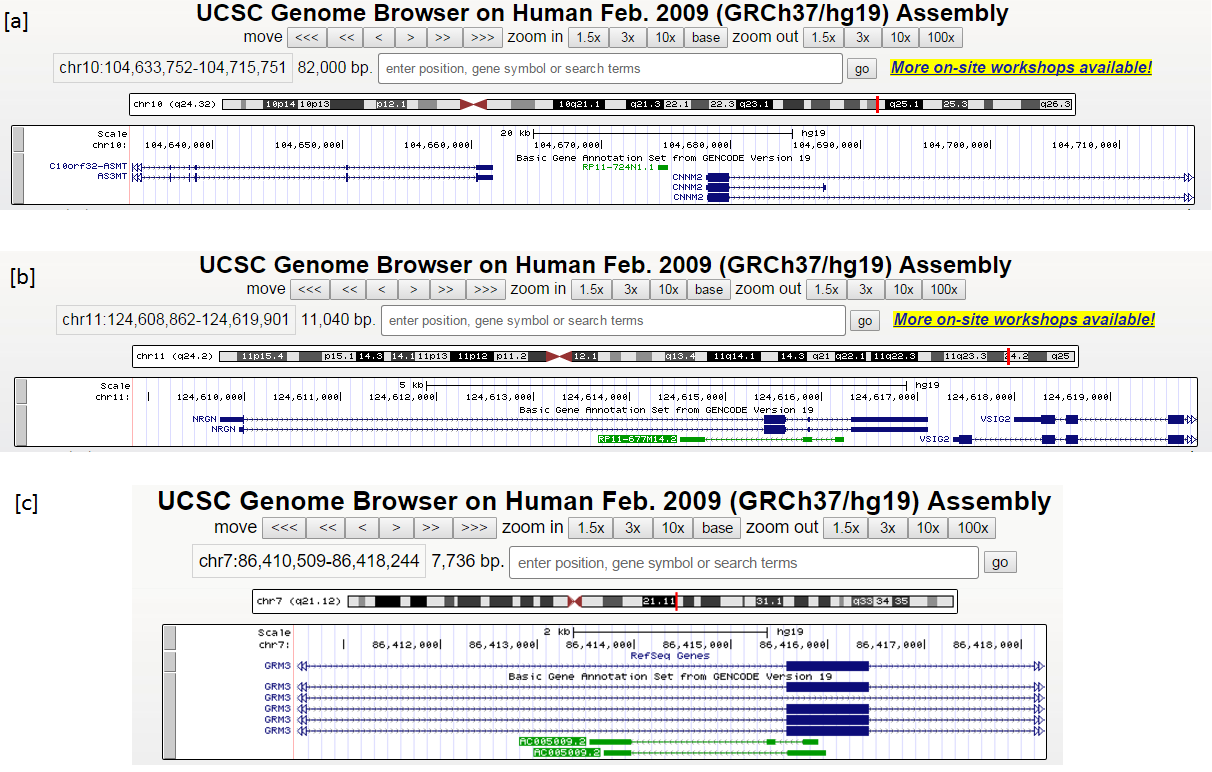

Supplement: Supplementary file 10 — Genome Browser Visualizations of lncRNAs and nearby genes [file 41398_2017_30_MOESM10_ESM.bmp]

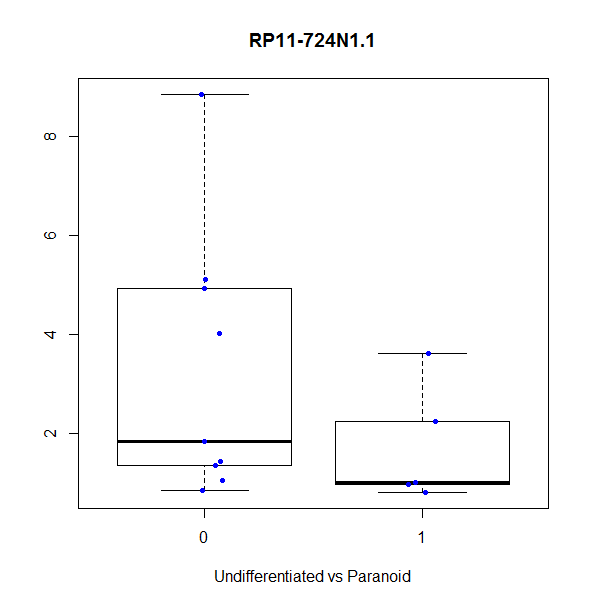

Supplement: Supplementary file 11 — Boxplot of RP11-724N1.1 between undifferentiated and paranoid [file 41398_2017_30_MOESM11_ESM.png]

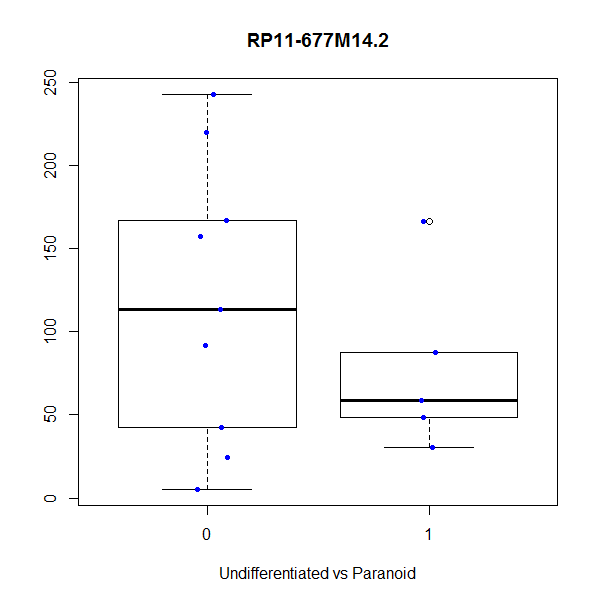

Supplement: Supplementary file 12 — Boxplot of RP11-677M14.2 between undifferentiated and paranoid [file 41398_2017_30_MOESM12_ESM.png]

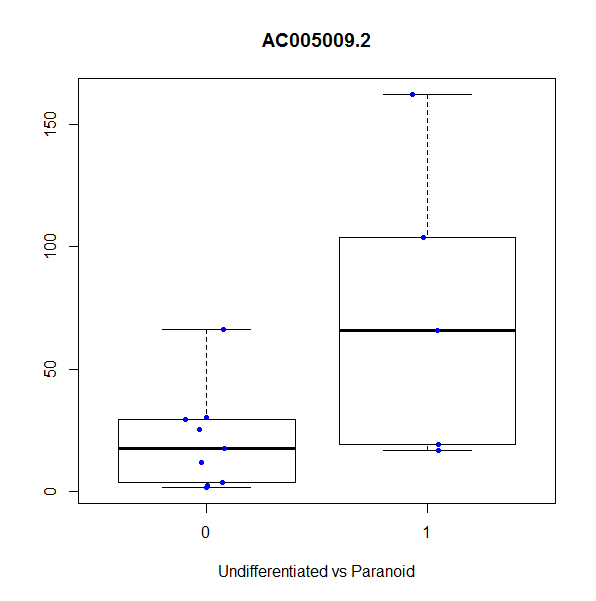

Supplement: Supplementary file 13 — Boxplot of AC005009.2 between undifferentiated and paranoid [file 41398_2017_30_MOESM13_ESM.png]

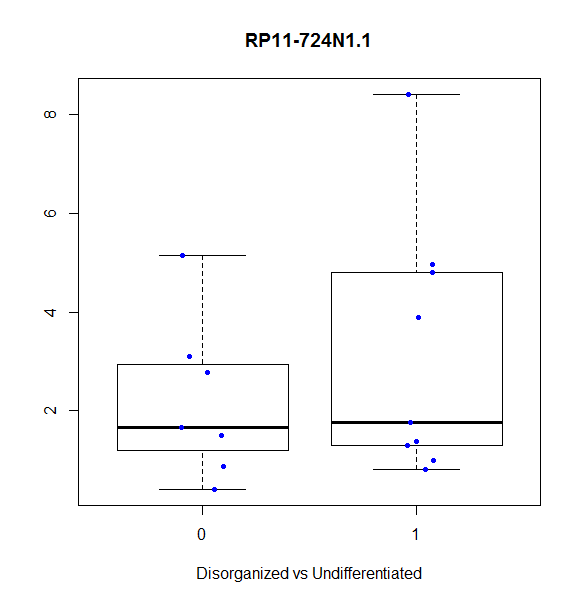

Supplement: Supplementary file 14 — Boxplot of RP11-724N1.1 between undifferentiated and disorganized [file 41398_2017_30_MOESM14_ESM.png]

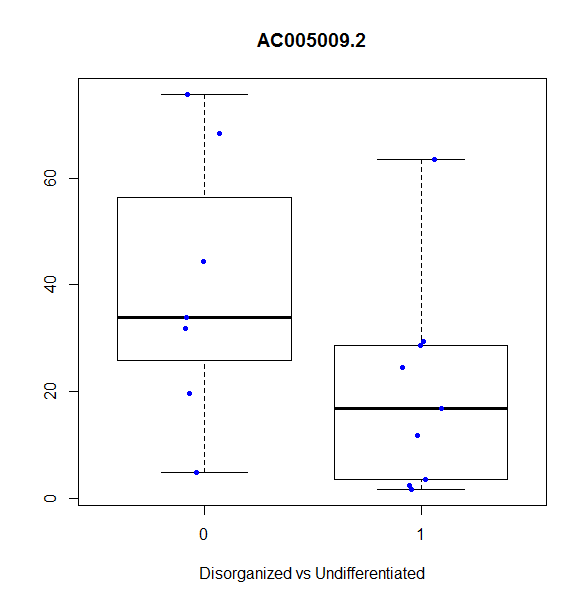

Supplement: Supplementary file 15 — Boxplot of AC005009.2 between disorganized and undifferentiated [file 41398_2017_30_MOESM15_ESM.png]

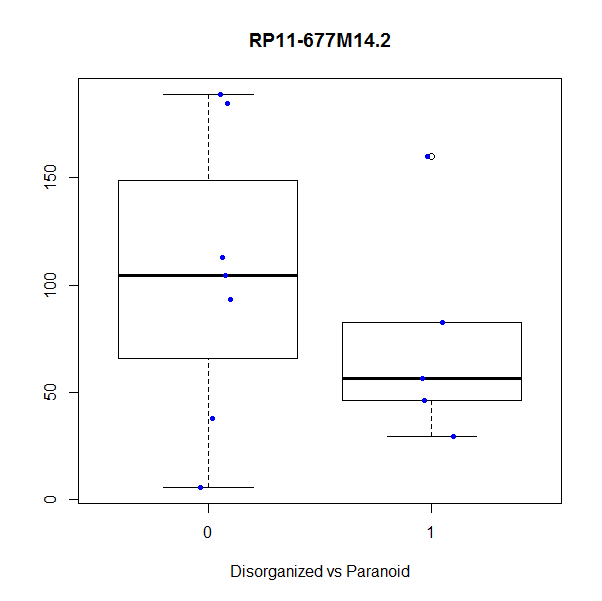

Supplement: Supplementary file 16 — Boxplot of RP11-677M14.2 between disorganized and paranoid [file 41398_2017_30_MOESM16_ESM.png]

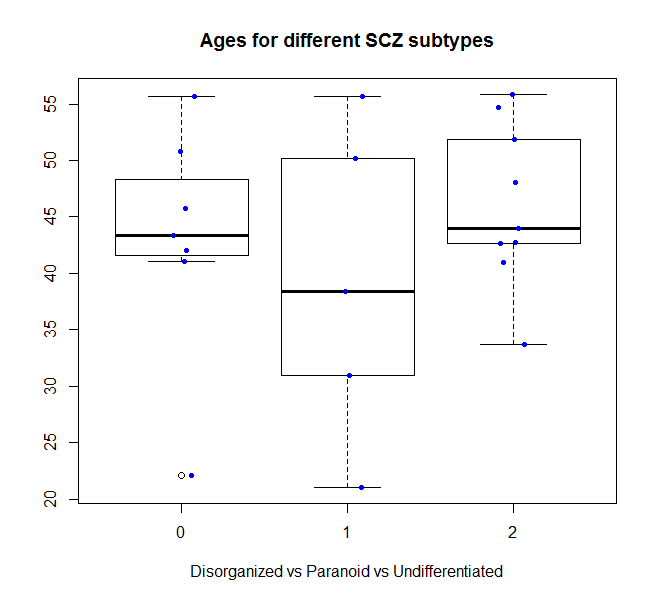

Supplement: Supplementary file 17 — Age distribution of SCZ subtype patients [file 41398_2017_30_MOESM17_ESM.png]

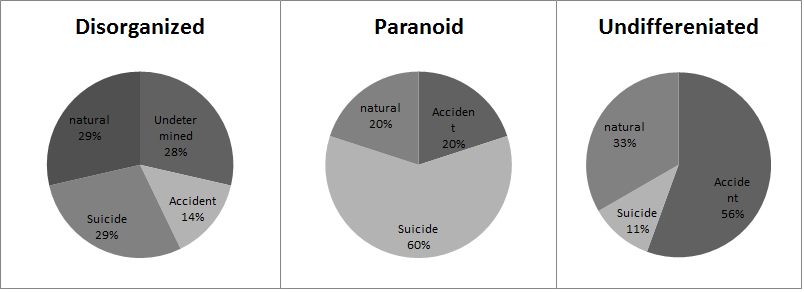

Supplement: Supplementary file 18 — Death distribution of SCZ subtype patients [file 41398_2017_30_MOESM18_ESM.png]
